# The effects of playing music on mental health outcomes

**DOI:** 10.1038/s41598-019-49099-9

**Published:** 2019-08-30

**Authors:** Laura W. Wesseldijk, Fredrik Ullén, Miriam A. Mosing

**Affiliations:** 1 Department of Neuroscience, Karolinska Institutet, Solnavägen 9, SE-171 77 Stockholm, Sweden; 2 Department of Psychiatry, Amsterdam UMC, University of Amsterdam, Meibergdreef 5, 1105 AZ Amsterdam, The Netherlands; 3 Department of Medical Epidemiology and Biostatistics, Karolinska Institutet, Nobels v 12A, 171 77 Stockholm, Sweden

**Keywords:** Behavioural genetics, Psychiatric disorders, Risk factors

## Abstract

The association between active musical engagement (as leisure activity or professionally) and mental health is still unclear, with earlier studies reporting contrasting findings. Here we tested whether musical engagement predicts (1) a diagnosis of depression, anxiety, schizophrenia, bipolar or stress-related disorders based on nationwide patient registers or (2) self-reported depressive, burnout and schizotypal symptoms in 10,776 Swedish twins. Information was available on the years individuals played an instrument, including their start and stop date if applicable, and their level of achievement. Survival analyses were used to test the effect of musical engagement on the incidence of psychiatric disorders. Regression analyses were applied for self-reported psychiatric symptoms. Additionally, we conducted co-twin control analyses to further explore the association while controlling for genetic and shared environmental confounding. Results showed that overall individuals playing a musical instrument (independent of their musical achievement) may have a somewhat increased risk for mental health problems, though only significant for self-reported mental health measures. When controlling for familial liability associations diminished, suggesting that the association is likely not due to a causal negative effect of playing music, but rather to shared underlying environmental or genetic factors influencing both musicianship and mental health problems.

## Introduction

The high suicide rate among famous musicians over the last few years, e.g. Soundgarden’s Chris Cornell, Linkin Park’s Chester Bennington, Avicii and the Prodigy’s Keith Flint, has received a lot of media attention and raised the question about a possible relationship between mental health problems and musicianship. In line with that, a recent survey among 2,211 British self-identified professional musicians found musicians to be up to three times more likely to report depressive problems than individuals in the general population^1^. Furthermore, several famous people engaged in creative professions other than musicianship were also known for their psychiatric illnesses, like Vincent van Gogh, Ernest Hemingway or John Nash. It has been shown that unaffected relatives of individuals with bipolar disorder or schizophrenia have higher levels of creativity^2–5^. Overall, such findings suggest that creativity and musicianship are risk factors for mental health problems.

On the other hand, there are many studies that report positive relationships between musical engagement and indicators of mental health, thus suggesting the opposite, namely that engagement in music could be protective against psychiatric problems. Although epidemiological studies investigating the association between music and the risk of mental health problems are rare – for a review, see^6^ – the few existing ones all tend to suggest a positive effect of music^7^. For example, singing or playing music has been reported to be have a positive influence on various subjective health outcomes, including anxiety and depression^8^. Singing in a choir is related to higher self-rated quality of life and satisfaction with health^9^, and playing an instrument, and singing or performing in theater, tend to be associated with increased self-rated health in women, but decreased all-cause mortality in men and not vice versa^10^. Hours of music practice has been shown to be associated with lower alexithymia (i.e., a dysfunction in emotional awareness, social attachment, and interpersonal relating)^11^. Finally, in 50,797 Norwegian males, but not in females, it was found that active participation in music, singing or theater predicted significantly lower depressive symptoms^12^. It is important to note that the measures of health outcomes in these studies are retrospective self-reports. Therefore, the outcomes could partly reflect characteristics of the rater and may be subject to a recall bias.

Furthermore, there are numerous reviews on the effect of music interventions, both active (e.g. performing) and passive (e.g. listening), on individuals in clinical settings, e.g. during medical procedures or in mental health clinics (for reviews in children, see^13–17^; for reviews in adults, see^18–20^). The majority of reviews conclude that music interventions have a positive effect on pain, mood, and anxious or depressive symptoms in both children and adults in clinical settings. This suggests not only a positive association in line with the epidemiological research, but also potentially a causal relationship. It is important to note that most of the music interventions described in these studies have been tailored to address individually assessed needs of a client by a music therapist, which differs significantly from self-initiated musical engagement in daily life. Furthermore, as pointed out in most of these reviews, it is difficult to draw firm conclusions about protective effect of music due to the mixed quality of many of the conducted studies, i.e., studies had small samples, suffered from bias due to methodological issues, and there was great variability among the results of the studies.

In sum, the direction of the association between musical engagement and mental health is still unclear with powerful population based research still failing to establish a relation unequivocally. Furthermore, it seems that differentiating between active amateur and professional musicians might explain the discrepancy between, on the one hand research reporting beneficial effects of music in everyday life on mental health, and on the other hand the high rate of depression and suicides among professional musicians. This view is in line with findings from the recent study of Bonde, *et al*.^21^ in which active professional musicians reported more health problems than active amateur musicians, while active amateur musicians reported significantly better self-reported health than non-musicians. Possibly, the strain and pressure experienced by professional musicians may override a possible overall positive effect of musical engagement. Furthermore, an association between engagement in music and mental health problems on a population level does not necessarily reflect causal effects; it could also reflect reverse causation or underlying shared genetic or shared environmental factors that influence both the choice to engage in music and the development of psychiatric problems. It is well known that genetic factors play a role both in mental health problems^22^ and in individual variation in music-related abilities^23^. In line with that, there is evidence that the association between creativity and psychiatric disorders is largely driven by underlying shared genetic factors^24^. Studying twins can reduce genetic and shared environmental confounding and strengthen causal inferences.

Here, using a large genetically informative sample of Swedish twins, we aim to investigate whether there is an association between active musical engagement defined by whether an individual plays an instrument, on an amateur and professional level, and mental health and if so, whether the relationship is consistent with a causal hypothesis, i.e., that musical engagement truly affects mental health. We use data from the Swedish nationwide in-patient and outpatient registers for psychiatric diagnoses (i.e., diagnosis of depression, anxiety disorder, schizophrenia, bipolar, stress disorder) as well as self-reports on mental health problems (depressive, burnout and schizotypal symptoms). As the association between playing sport and mental health is already well established, we conducted sensitivity analyses investigating a protective effect of sport against psychiatric problems in this sample.

## Methods

### Participants

Data for the present study was collected as part of “the Study of Twin Adults: Genes and Environment” (STAGE), a sub-study in a cohort of approximately 32,000 adult twins registered with the Swedish Twin Register (STR). The STAGE study sent out a web survey in 2012–2013 inquiring about, musical engagement and musical achievement and other potentially music related traits. The 11,543 responders were aged between 27 and 54 years and data were available for 10,776 individuals on musical engagement and for 6,833 on musical achievement.

The National Patient Register (NPR) records the use of the health care system in Sweden, which has nationwide coverage ensuring equal access to health care for all residents, using a 10-digit personal identification number assigned to all Swedish residents^25^. The NPR includes an in-patient register (IPR) and out-patient register (OPR). The IPR contains information about hospitalizations since 1964 (with full national coverage since 1977), while the OPR covers outpatient visits since 2001^26^. The Cause of Death Register (CDR) contains information from death records since 1961^27^. The Swedish twins from the STAGE study were linked to records from the IPR, OPR and CDR.

Informed consent was obtained from all participants. The study was approved by the Regional Ethics Review Board in Stockholm (Dnr 2011/570-31/5, 2012/1107-32, 2018/866-32). All research methods were performed in accordance with relevant guidelines and regulations.

### Measures

#### Musical engagement

Participants were asked whether they ever played an instrument. Those who responded positively were asked at what age they started to play, whether they still played an instrument and, if not, at what age they stopped playing. From these questions, a music status variable was created (0: does not play, 1: used to play, 2: plays).

#### Sport engagement

Participants reported on whether they ever actively trained a sport (excluding exercise training or physical activity in general). Information on age they started training a sport, whether they still played and at what age they stopped playing resulted in a sport status variable with 0 ‘does not play’, 1 ‘used to play’ and 2 ‘plays sport’.

#### Musical achievement

Musical achievement was measured with a Swedish version of the Creative Achievement Questionnaire (CAQ) that assesses different domains of creativity, including music^28,29^. Individuals were asked to rate their musical achievement on a seven-point scale: 1 ‘I am not engaged in music at all’, 2’I have played or sang privately, but I have never played, sang or showed my music to others’, 3’I have taken music lessons, but I have never played, sang or showed my music to others’, 4 ‘I have played or sung, or my music has been played in public concerts in my home town, but I have not been paid for this’, 5 ‘I have played or sung, or my music has been played in public concerts in my home town, and I have been paid for this’, 6 ‘I am professionally active as a musician’ and 7 ‘I am professionally active as a musician and have been reviewed/featured in national or international media and/or have received an award for my musical activities’. To differentiate between amateur and professional musicians, we converted the scale to three groups: 1 ‘no engagement in music’, 2–4 ‘making music on an amateur level’, and 5–7 ‘professionally active in music’.

#### Registry-based mental health outcomes

For each individual we derived information (diagnosis and date of first diagnosis) on incidence of depression, anxiety disorder, schizophrenia, bipolar disorder, or stress disorder based on clinical diagnoses after any inpatient or outpatient visit, or underlying cause of death registered in the national registers according to *the International Classification of Diseases* (ICD) codes as reported in Table 1. We created an ‘any psychiatric diagnosis’ variable indicating whether the participant has ever been diagnosed with any of the five categories of clinical diagnoses above. For this variable, we selected the earliest date of diagnosis in case of comorbidity.

**Table 1:** International Classification of Diseases (ICD) codes for the included mental health outcomes.

	ICD 7 (1964–1968)	ICD 8 (1969–1986)	ICD 9 (1987–1996)	ICD 10 (1997–2015)
Depression	301.1, 314, 790.2	296.2, 300.4	296B, 300E, 311	F32-F39
Anxiety	310.99, 311.99, 312.99	300 excluding 300.4	300 excluding 300E	F40-F4
Schizophrenia	300, 300.6	295.0–295.4, 295.6, 295.8, 295.9	295A-295E, 295G, 295H, 295W, 295X	F20, F25
Bipolar	301.0	296.0–296.3, 296.8, 296.9	296A-296E, 296W, 296X	F30, F31
Stress- related	—	307, 308.4	308, 309	F43

#### Questionnaire-based self-reported mental health

In addition, self-reports on mental health outcomes (i.e., depressive, burnout and schizotypal symptoms) obtained in the web survey were analyzed. *Depressive symptoms* were measured with the depression scale of the Hopkins Symptom Checklist^30^. This scale contains of six items all ranging from 0 to 4 (0 ‘not at all’ to 4 ‘extremely’), measuring depressive symptoms in a work-related context, with higher scores indicating more depressive symptoms. *Burn-out symptoms* related to work were measured with the Emotional exhaustion subscale of the Maslach Burnout Inventory-General Survey^31^. This scale consists of five items that range from 1 (every day) to 6 (a few times per year or less/never). Therefore, as higher scores reflect less burnout symptoms, we reversed this scale so that higher scores indicate more burnout symptoms in line with the other mental health outcomes. *Schizotypal symptoms* were measured with the “Positive Dimension Frequency Scale” of the Community Assessment of Psychic Experiences (CAPE) questionnaire^32^. The score is based on 20 positive symptom items that can be answered with four different symptom frequency levels, from 1 ‘never’ to 4 ‘almost always’. Higher scores indicate more schizotypal symptoms. The Cronbach alpha reliability in present study was 0.89 for the depressive symptom scale, 0.87 for the burnout symptom scale and 0.79 for the schizotypal symptom scale.

#### Level of education

Educational achievement was dichotomized into ‘low and intermediate’ (1 to 7; unfinished primary school to bachelor education) and ‘high’ (8 to 10; master education to PhD).

### Statistical analyses

All analyses were conducted in STATA 15.

#### Registry-based mental health outcomes

*Survival analyses*, i.e., Cox proportional hazard regression, were conducted to explore the effect of musical engagement and musical achievement on the risk to receive a registry-based diagnosis of a psychiatric disorder^33^. Survival analysis is a method to analyze data where the outcome variable is the time until an event happens. The time (years) from the age of twelve to either the date of first receiving a psychiatric diagnosis or to the date of censoring (i.e., date of death or end of follow-up at January 1, 2015) were used as the time scale (i.e., the survival time). For the analyses on the effect of *musical engagement*, we had to take into account that some individuals had not yet started playing an instrument at the age of twelve (i.e., would start at a later age), or stopped playing at some stage. Therefore, years were split on whether the individual did not play, stopped or started playing, or currently played a musical instrument using the *stsplit* statement to differentiate between the three levels of musical engagement. We used Cox proportional hazard regressions, a method that assumes the effect upon survival to be constant over time, to calculate hazard ratios (HRs) with 95% confidence intervals. The HRs represent the effects of 1) playing an instrument versus never having played an instrument or 2) having played an instrument (but stopped before diagnosis) versus never having played an instrument on the baseline risk for a mental health diagnosis (independent of playing status) during the follow-up period. A HR value greater than one indicates an increased risk, while a value below one indicates a protective effect. Additionally, we conducted the survival analyses to estimate the effect of *musical achievement* in a lifetime on the risk of a mental health diagnosis, in which the HRs represent 1) the effect of having performed music as an amateur versus not being involved in music, or 2) the effect of having performed music professionally versus not being involved in music. As we analyzed the three level musical achievement in a lifetime, we did not split years on age (assuming that individuals have been on a lifelong ‘achievement’ trajectory). To correct for relatedness in the twin sample, the robust standard error estimator for clustered observations was used^34^. We fitted separate survival models for each of the five psychiatric disorder diagnoses as well as for the ‘any psychiatric diagnosis’ variable. Thus, first, we in total fitted six models for the effect of musical engagement and another six models for musical achievement. All models included sex as a covariate. Additionally, we fitted all models corrected for level of education, resulting in a small loss of data due to missing information for some individuals, therefore reducing the power. For each model, the proportional hazards assumption was tested using Schoenfeld residuals. No evidence for deviation from the proportional hazards assumption was found for any of the models (all p values > 0.01). As a sensitivity analysis, the above-described models for musical engagement (in which we used the *stsplit* statement) were repeated with *sport engagement* as the exposure variable instead, to estimate the effect of playing sport on registry-based psychiatric disorder diagnoses.

#### Self-reported mental health outcomes

*Linear regression analyses* were performed to explore the effect of musical engagement and musical achievement on the self-rated continuous measures of depressive symptoms, burnout symptoms and schizotypal symptoms. To correct for relatedness in the twin sample, we used the robust standard error estimator for clustered observations. We included sex as a covariate. Additionally, we ran the analyses corrected for level of education. As a sensitivity analysis, we estimated the effect of sport engagement on depressive, burnout and schizotypal symptoms using linear regression analyses.

#### Co-twin control analyses (within-pair analyses)

*Within-pair analyses in identical twins* were conducted to further explore the association between musical engagement and receiving a mental health diagnosis when controlling for genetic and shared environmental factors. As monozygotic (MZ) twins are genetically identical and share their family environment, studying identical twins excludes confounding in case a genetic predisposition or shared environmental influence affects both outcome (mental health problems), and exposure (music engagement). Therefore, if music engagement truly causes a lower/higher risk for receiving a mental health diagnosis, we would expect the MZ twin that plays music to have a lower/higher risk of psychiatric problems than his or her co-twin that does not play music. Conditional Cox regression models, with the *strata* statement to stratify by pair identifier, were fitted for the mental health diagnoses to estimate HRs with 95% confidence intervals. Notably, only complete identical twin pairs discordant for exposure (i.e., music engagement) and outcome (i.e., the psychiatric disorder diagnosis) contribute to the within-pair analyses. The conditional logistic regression estimates the effect of the difference between the two observations in the strata. Twins are regarded as discordant for the outcome when the time of the psychiatric diagnosis differs. Due to the low prevalence of schizophrenia and bipolar disorder in the complete twin pairs, these phenotypes were excluded from the within-pair analyses.

Additionally, to explore further the effect of music engagement on the self-rated continuous measures of depressive symptoms, burnout symptoms and schizotypal symptoms, we conducted within-pair linear regression analyses using the *xtreg fe* statement to stratify by twin pair. In within-pair analyses in identical twins correcting for sex is not required as each twin is matched to his or her co-twin. To increase power, we also included data from same-sex dizygotic (DZ) twins (who share on average 50% of their genetic makeup and 100% of their family environment).

## Results

### Descriptives

Information on mental health outcomes and musical engagement was available for 9,816 individuals [2,212 complete twin pairs (1,055 MZ, 661 dizygotic same-sex (DZ), 496 dizygotic opposite-sex (DOS) twins) and 5,392 individual twins]. Among these individuals, data on musical achievement were available for 6,295 individuals [1,208 complete twin pairs (627 MZ, 342 DZ, 239 DOS) and 3,879 individual twins]. Characteristics of the participants are reported in Table 2.

**Table 2:** Sample characteristics.

	Musical engagement	Musical achievement
	N individuals (%)	N individuals (%)
MZm	1,466	948
MZf	2,287	1,526
DZm	1,227	783
DZf	1,628	1,038
DOS	2,903	1,800
Unknown	305	200
Mean age	40.73 (*SD* 7.74)	40.90 (*SD* 7.84)
Education		
*low*	3,592 (47.7%)	2,948 (46.8%)
*high*	3,962 (52.4%)	3,347 (53.2%)
Any psychiatric disorder	831 (8.5%)	531 (8.4%)
Depression	482 (4.9%)	315 (5%)
Anxiety	408 (4.2%)	245 (3.9%)
Schizophrenia	24 (0.2%)	19 (0.3%)
Bipolar	86 (0.9%)	50 (0.8%)
Stress-related	271 (2.8%)	171 (2.7%)

Women were more likely to initiate playing an instrument than men (37.7% of men versus 20.5% of women), while roughly the same amount of men and women remained actively involved in music in adulthood (23.3% of men and 21.9% of women). More men (8.5%) than women (5%) played music professionally.

### Registry-based mental health outcomes

**Figure 1:**
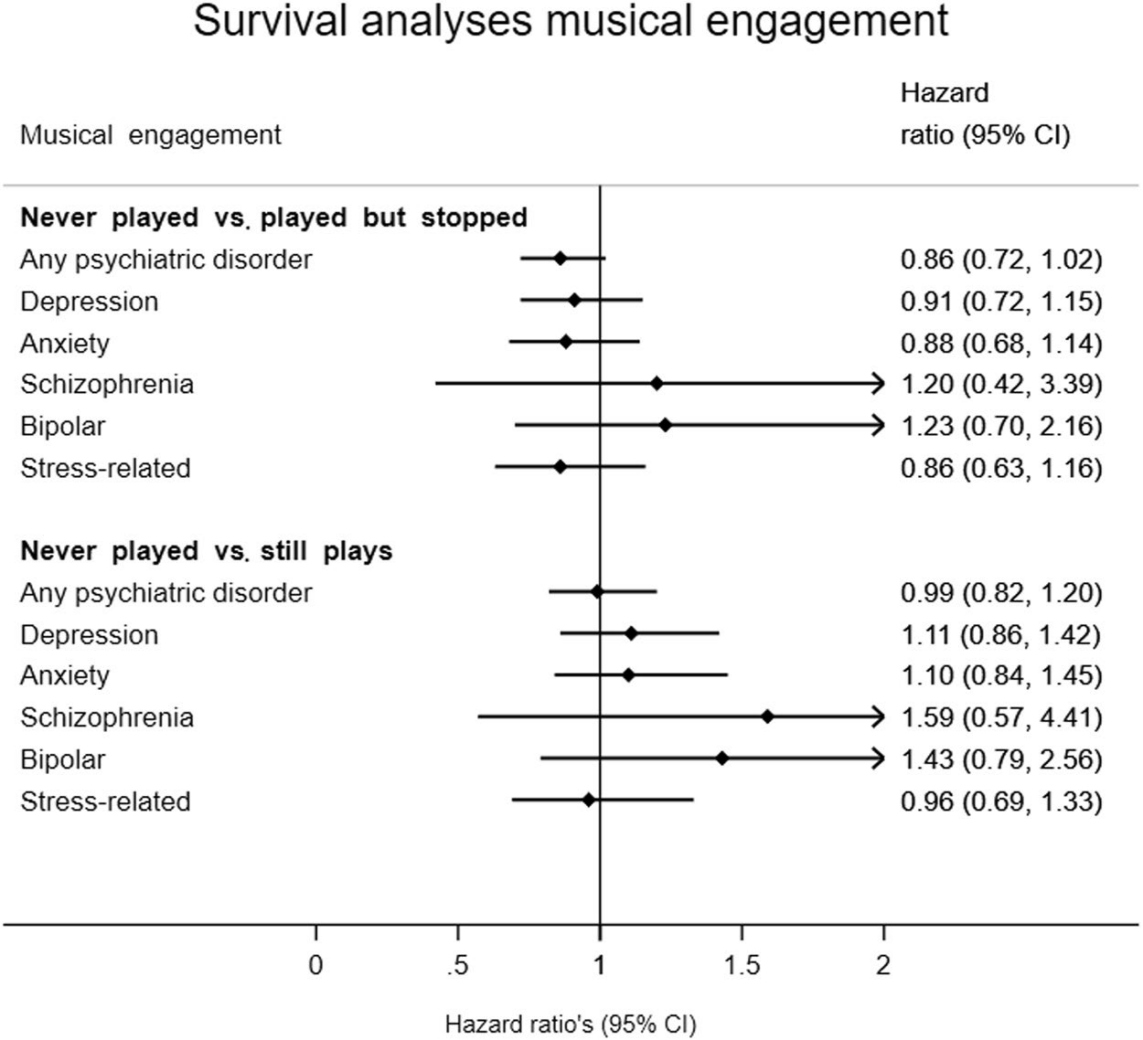
Music engagement and registry-based mental health outcomes. Sex is included as covariate.

**Figure 2:**
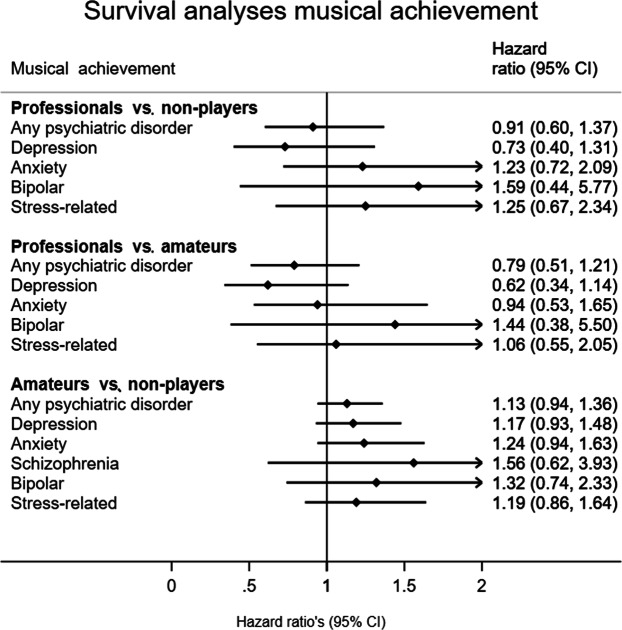
Music achievement and registry-based mental health outcomes. Sex is included as a covariate.

Although overall, there was an overall trend towards a somewhat elevated risk for psychiatric disease in those engaged with music, neither playing music nor having played music in the past (Fig. 1), nor professional musicianship (Fig. 2) was significantly associated with the risk for any of the psychiatric disorders. The analyses adjusted for level of education showed similar results (see Table S1 for musical engagement and Table S2 for musical achievement, in the supplementary material), with the exception that individuals who played an instrument had a significantly higher risk (39%) of being diagnosed with an anxiety disorder (HR 1.39, CI 1.01–1.92) compared to those who never played an instrument. In terms of covariates, we found females to have a higher risk for depression (92%), anxiety disorder (92%), and stress-related disorders (58%) (Table S1). Additionally, individuals with higher levels of education had a significantly lower risk for psychiatric disorders, depression, anxiety disorder, schizophrenia or bipolar disorder (Table S1).

### Self-reported mental health

Results of the regression analyses with self-reported mental health symptoms indicated that playing an instrument was significantly associated with more schizotypal symptoms and depressive and burnout symptoms in a work context (see left part of Table 3). Having played an instrument in the past did not significantly influence any of the self-rated mental health outcomes. Furthermore, even though professional and amateur musicians report more burnout and schizotypal symptoms than non-players, individuals who played music professionally did not experience significantly more depressive, burnout or schizotypal symptoms than individuals who play music on an amateur level (see right part of Table 3). When analyses were repeated adjusting for level of education (results not shown) all results remained the same.

**Table 3:** The effect of playing music and of musical achievement on the self-reported mental health outcomes. Sex is included as a covariate.

	Playing music						Musical achievement								
	Never played vs. played but stopped			Never played vs. still plays			Professional musicians vs. non-players			Professional musicians vs. amateur musicians			Amateur musicians vs. non-players		
	*β*	*SE*	*p-value*	*β*	*SE*	*p-value*	*β*	*SE*	*p-value*	*β*	*SE*	*p-value*	*β*	*SE*	*p-value*
Depressive symptoms	0.05	0.03	0.08	**0.09**	**0.03**	**0.01**	0.13	0.05	0.02	0.05	0.05	0.38	0.06	0.03	0.02
Burnout symptoms	0.05	0.03	0.07	**0.13**	**0.03**	**<0.001**	**0.18**	**0.05**	**0.001**	0.08	0.06	0.14	**0.08**	**0.03**	**<0.01**
Schizotypal symptoms	0.04	0.03	0.24	**0.12**	**0.04**	**0.001**	**0.24**	**0.06**	**<0.001**	0.15	0.07	0.02	**0.09**	**0.03**	**0.001**

### Sensitivity sport analyses

Results of the sensitivity analyses on the registry-based mental health outcomes showed that individuals who actively played sports were less likely to develop any psychiatric disorder, as well as depression, anxiety, and bipolar disorder (see Fig. S1). There was no sustained beneficial effect of past sports engagement after stopping with exercise. The analyses adjusted for level of education showed the same results.

Regression analyses on the self-reported mental health outcomes showed that individuals who actively play sports were significantly less likely to report depressive symptoms (β = −0.23, p < 0.001) and burnout symptoms (β = −0.20, p < 0.001), but not schizotypal symptoms (β = 0.00, p = 0.96). Past sport activities were unrelated to the self-reported mental health outcomes (p values range between 0.08 and 0.20). Including level of education in the analyses did not affect the results.

### Co-twin control analyses

Results of the co-twin control analyses for both the registry-based and self-reported mental health measures are shown in Table 4. None of the within-pair estimates were significant. However, overall, the effect sizes (HR or beta) moved closer to zero with increased controlling of shared liability.

**Table 4:** Co-twin control analyses within monozygotic (MZ) as well as both MZ and dizygotic (DZ) twin pairs.

	MZ							MZ + DZ						
	N	Never played vs. played but stopped			Never played vs. still plays			N	Never played vs. played but stopped			Never played vs. still plays		
Any psychiatric disorder	27	0.55 (0.21–1.47)			0.63 (0.22–1.75)			56	0.89 (0.47–1.66)			0.95 (0.49–1.83)		
Depression	16	0.55 (0.17–1.79)			0.76 (0.21–2.62)			31	0.78 (0.34–1.78)			0.97 (0.41–2.29)		
Anxiety	18	0.32 (0.07–1.42)			0.51 (0.12–2.05)			32	0.87 (0.33–2.31)			1.20 (0.43–3.27)		
Stress-related	6	0.52 (0.05–4.94)			1.23 (0.16–9.32)			24	1.43 (0.49–4.12)			1.70 (0.61–4.76)		
		***β***	***SE***	***p-value***	***β***	***SE***	***p-value***		***β***	***SE***	***p-value***	***β***	***SE***	***p-value***
Depressive symptoms	744	−0.01	0.11	0.94	−0.00	0.14	0.98	1,135	0.02	0.09	0.85	0.07	0.11	0.56
Burnout symptoms	747	0.06	0.11	0.59	−0.05	0.14	0.73	1,138	−0.01	0.08	0.95	−0.02	0.11	0.87
Schizotypal symptoms	621	−0.06	0.11	0.59	−0.12	0.14	0.38	924	−0.06	0.09	0.48	−0.12	0.12	0.31

The upper part of the table shows the hazard ratios (confidence intervals) for the registry-based mental health outcomes; the lower part reports the results from the within-pair regression analyses on the self-reported mental health outcomes.

## Discussion

We aimed to investigate the association between musical engagement in everyday life and mental health in a large cohort of Swedish twins. Although the findings were somewhat mixed, overall results suggest that individuals who actively play a musical instrument (but not necessarily professionally) may have a somewhat increased risk for mental health problems. However, when controlling for familial liability these associations became weaker and non-significant suggesting that the association is likely explained by underlying shared factors influencing both musicianship and mental health problems.

While analyses using registry-based mental health diagnoses showed no significant association between music playing or professional musical engagement and psychiatric diagnoses, the direction of the effect was trending towards a somewhat increased risk for psychiatric diagnoses for those actively engaged with music. Results from the self-reported mental health outcomes further supported this; individuals playing an instrument report more depressive, burnout and schizotypal symptoms. This is in contrast with previous epidemiological and clinical studies reporting positive effects of musical engagement on anxious and depressive symptoms^6–20^. Further, a recent study by Fancourt and Steptoe^35^ found cultural engagement to decrease the development of depression in older ages. However, it appears likely that it is important to distinguish between general cultural engagement, i.e., visits to the theatre, concerts or opera, the cinema or an art gallery, exhibition or museum) and active playing of a musical instrument, which is the focus of the present study. Playing a musical instrument is much more narrowly defined behavior and involves many (cognitive and physical) processes different from engaging in cultural musical activities or listening to music. On the other hand, our findings are in line with results from the survey among British professional musicians^1^ and with previous findings of associations between creativity and mental health problems, i.e., that people engaging in creative activities tend to experience more psychiatric problems^4^. It is important to note that the previous epidemiological studies on mental health, the British musicians study, but also our continuous mental health outcomes, were based on self-report. An explanation could be that results of self-report reflect a different attitude towards mental health among more creative individuals, with higher acceptance and awareness of mental health problems, possibly resulting in over-reporting in the field.

Further, there is evidence that the association between creativity and psychiatric disorders can be largely attributed to underlying shared genetic factors^24,36^. This is in line with present results of our co-twin control analyses, which showed that the association between musicianship and mental health was attenuated when controlling for genetic and shared environmental confounding (although all analyses were non-significant). This suggests that the observed associations would partly be explained by a shared underlying etiology, (i.e., genetic or family environmental factors which affect both, individuals differences in music playing and mental health) and not by a causal effect of playing music. The within-pair results, however, should be interpreted with caution as only discordant twin pairs contribute to the co-twin control analyses, which reduced the power to find significant associations.

We found significant differences between professional or amateur musicians and non-players in self-rated health outcomes, which are in line with our findings on playing music in general. However, in neither self-rated nor registry-based data, we observed any significant differences in mental health problems between professional musicians compared to amateur musicians. This is in contrast to findings from the study of Bonde, *et al*.^21^ in which active professional musicians reported higher numbers of overall health problems than active amateur musicians, while active amateur musicians reported significantly better self-reported health than non-musicians did. Whilst this was also a large population-based sample, this study analyzed general health instead of mental health, which likely contributes to the difference in findings.

The discrepancy in findings between registry-based mental health diagnoses and self-reported mental health could be due to an influence of rater and recall biases captured in the self-reported mental health outcomes, as discussed above. However, another explanation could be less power in the analyses with the registry-based mental health diagnoses to detect an existing effect. The power of a method to analyze survival time data depends partly on the number of psychiatric diagnoses rather than on the total sample size. In the present sample, observed post-hoc power for the survival analyses to detect a HR of 0.8 for music engagement is 88% for the incidence of a psychiatric disorder, 67% for depression, 61% for anxiety, 7% for schizophrenia, 18% for bipolar and 45% for stress disorder, reflecting the different incident rates of the disorders. As the self-reported mental health problems were measured on a continuous scale, these analyses have higher power (i.e., no cut-off score needs to be reached to obtain a full diagnosis). Nevertheless, our sensitivity analyses in the registry-based outcomes on the effect of sport did show a significant protective effect of sport against the risk of receiving a diagnosis of a psychiatric disorder, depression, anxiety and bipolar disorder in this sample, suggesting that an association can be found with the present distribution of the data if existent. Therefore, we conclude that a lack of power is not a likely explanation for our null findings in the registry-based health outcomes, and that if there truly were an effect, it would be very small.

There are some limitations of this study in addition to the ones we already touched upon. We analyzed data on psychiatric diagnoses obtained from the Swedish nationwide in-patient and outpatient registers. However, the outpatient register only reached full coverage in 2001 and it is therefore possible that some individuals were not classified with a psychiatric disorder, although they did experience mental health problems before 2001. The same holds for individuals with mental health problems who did not visit a doctor. In addition, the dichotomous rather than dimensional nature of psychiatric diagnoses excludes large parts of the continuous variation among individuals in psychiatric problems. The continuous symptom scales increase the power to detect an effect of engagement in music or sports, but may be somewhat biased. Furthermore, our study explored potential effects of active musical engagement (i.e., making music) in everyday life and therefore our findings do not allow for any conclusions about the potential effect of (personalized) musical interventions on mental health problems. Lastly, as mentioned earlier, the sample of discordant twin pairs contributing to the co-twin control analyses was small, resulting in low power to detect effects.

To our knowledge, the present population-based study is the only genetically informative large-scale study to investigate associations between active engagement in music (both as a leisure activity and professionally) and registry-based as well as self-reported mental health outcomes. Rather than a protective effect of music engagement in everyday life as often suggested, our findings suggest that individuals actively engaged in music playing, but not only professional musicians, may have a somewhat elevated risk for mental health problems. This association may at least partly be due to shared underlying etiology and it is unlikely that it reflects a causal effect of playing music.

## Supplementary information

Supplementary Tables
