# Supplementary material for: The effects of playing music on mental health outcomes
Source: Sci Rep. 2019 Aug 30;9:12606. doi: 10.1038/s41598-019-49099-9 (PMC6717192; doi:10.1038/s41598-019-49099-9)

The effects of playing music on mental health outcomes
Laura W. Wesseldijk, Fredrik Ullén, Miriam A. Mosing
Supplementary material

Table S1. Music engagement and the registry-based mental health outcomes, including education and sex as covariates.

	Hazard ratio (confidence intervals)			
	Never played vs. played but stopped	Never played vs. still plays	Education	Sex
Any psychiatric disorder	0.88 (.72-1.08)	1.09 (.87-1.36)	**0.77 (.64-.91)**	**1.77 (1.47-2.13)**
Depression	0.91 (.69-1.19)	1.16 (.87-1.56)	**0.73 (.58-.92)**	**2.03 (1.59-2.59)**
Anxiety	0.95 (.70-1.29)	**1.39 (1.01-1.92)**	**0.59 (.46-.75)**	**2.03 (1.53-2.68)**
Schizophrenia	1.59 (.52-4.88)	2.42 (.78-7.43)	**0.27 (.09-.77)**	0.61 (.25-1.49)
Bipolar	1.23 (.63-2.37)	1.79 (.92-3.48)	0**.57 (.34-.94)**	1.40 (.82-2.39)
Stress-related	0.91 (.64-1.30)	1.00 (.69-1.47)	0.89 (.67-1.18)	**1.67 (1.21-2.28)**

Table S2. Music achievement and the registry-based mental health outcomes. Education and sex are included as covariates, but not reported in this table, see for their effects Table S1.

	Hazard ratio (confidence intervals)		
	Professional musicians vs. non-players	Professional musicians vs. amateur musicians	Amateur musicians vs. non-players
Any psychiatric disorder	.95 (.63-1.45)	.84 (.54-1.28)	1.17 (.96-1.41)
Depression	.77 (.42-1.40)	.66 (.36-1.22)	1.21 (.95-1.54)
Anxiety	1.37 (.79-2.34)	1.01 (.57-1.77)	1.32 (.99-1.73)
Schizophrenia	-	-	1.76 (.70-4.40)
Bipolar	1.73 (.46-6.46)	1.56 (.40-6.00)	1.38 (.77-2.46)
Stress-related	1.27 (.67-2.40)	1.11 (.58-2.13)	1.20 (.86-1.67)

Figure S1. Sensitivity analyses of the effect of sport on the registry-based mental health outcomes.

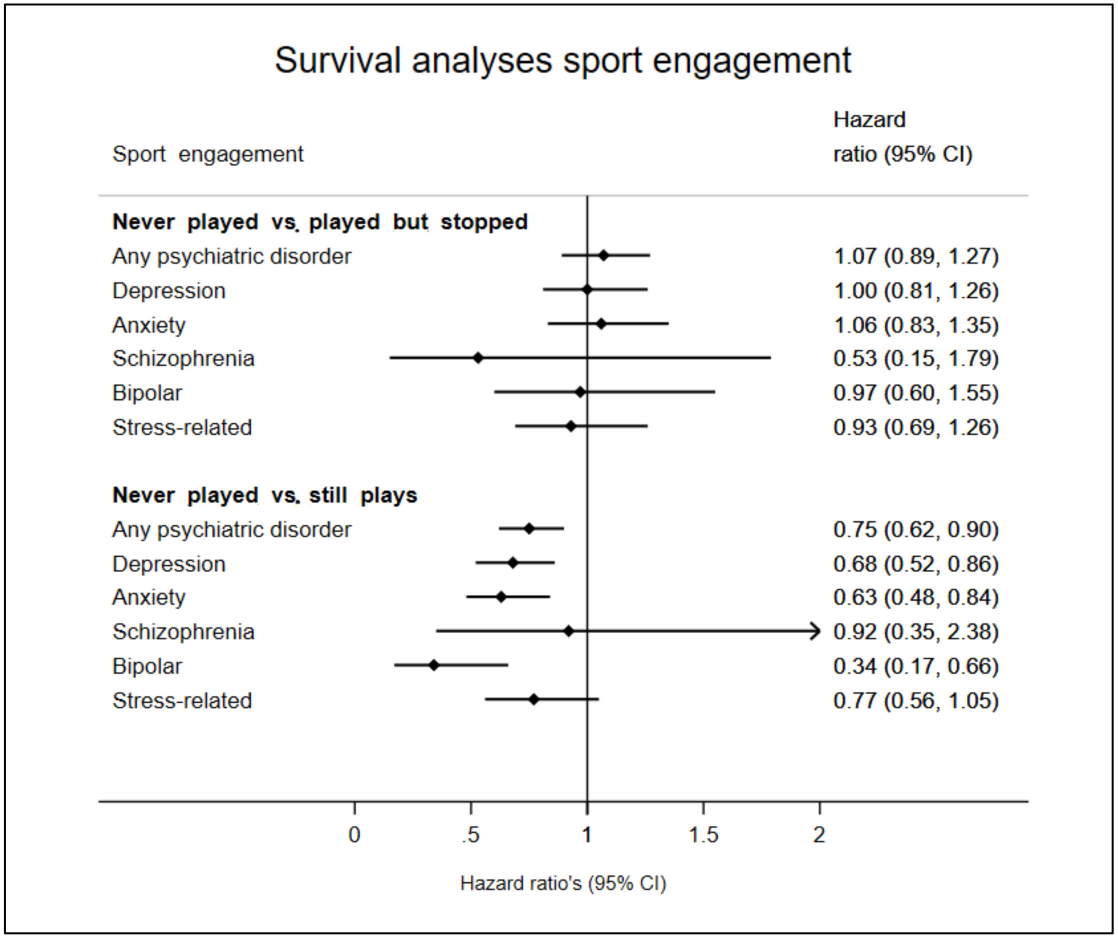

Supplement: Supplementary file 1 — Supplementary Tables [file 41598_2019_49099_MOESM1_ESM.pdf]
